# 3D Cardiac Constructs in Drug Discovery: Current Advances and Future Challenges

**DOI:** 10.34133/research.1165

**Published:** 2026-03-03

**Authors:** Chang Liu, Jing Guo, Gunash Mirzayeva, Michail Spanos, Ruoting Teng, Guoping Li, Dragos Cretoiu, Zhaoyang Chen, Junjie Xiao

**Affiliations:** ^1^ Cardiac Regeneration and Ageing Lab, Institute of Geriatrics (Shanghai University), Affiliated Nantong Hospital of Shanghai University (The Sixth People’s Hospital of Nantong) and School of Life Science, Shanghai University, Nantong 226011, China.; ^2^ Institute of Cardiovascular Sciences, Shanghai Engineering Research Center of Organ Repair, Joint International Research Laboratory of Biomaterials and Biotechnology in Organ Repair (Ministry of Education), School of Life Science, Shanghai University, Shanghai 200444, China.; ^3^ Cardiovascular Division of the Massachusetts General Hospital and Harvard Medical School, Boston, MA 02114, USA.; ^4^ Department of Internal Medicine, Albert Einstein College of Medicine, NCB, Bronx, NY, USA.; ^5^ Department of Medical Genetics, Carol Davila University of Medicine and Pharmacy, Bucharest 020031, Romania.; ^6^ Materno-Fetal Assistance Excellence Unit, Alessandrescu-Rusescu National Institute for Mother and Child Health, Bucharest 011062, Romania.; ^7^ Department of Cardiology, Heart Center of Fujian Province, Fujian Medical University Union Hospital, Fuzhou, Fujian 350001, China.

## Abstract

Advances in 3-dimensional (3D) cardiac constructs have provided powerful preclinical models for investigating the pathological mechanisms underlying human cardiovascular diseases and facilitating drug discovery. In this review, we focused on recent advancements in 3D cardiac constructs technology and explored its potential applications in drug development. We summarized the main types of disease models built on 3D cardiac constructs, the key parameters used for assessing pathology and drug efficacy, and their role in drug discovery. We also discussed the application of novel biomaterials in 3D cardiac constructs, the latest research progress in 3D cardiac constructs-based drug screening, and the transformative potential of artificial intelligence-assisted research on 3D cardiac constructs. Finally, we addressed the existing technical limitations and outline future directions for the development of 3D cardiac constructs.

## Introduction

Cardiovascular diseases (CVDs) are one of the major causes of global mortality and remain a marked challenge for public health. CVDs encompass various clinical manifestations, including myocardial infarction (MI), congenital heart disease, hypertension, and arrhythmias, among others [1]. Currently, rodent models are commonly utilized in CVD research; however, due to genetic differences between rodents and humans, human disease phenotypes cannot be fully recapitulated in rodent models. As a result, clinically relevant models, such as organoid models derived from human pluripotent stem cells (hPSCs), are necessary for elucidating CVD pathogenesis and advancing drug discovery.

Three-dimensional (3D) cardiac constructs are in vitro models typically derived from pluripotent stem cells and cultured with specialized structural scaffolds or in suspension systems [2]. These 3D constructs provide a unique and invaluable opportunity to study diseases as they morphologically and physiologically recapitulate human hearts. This is particularly important in the context of cardiac diseases, given the unique characteristics of human heart. The term “3D cardiac constructs” has been widely applied in research through various techniques. By offering physiologically relevant and highly customizable platforms, 3D cardiac constructs bridge critical gaps in traditional cardiac research methods and provide transformative insights into the development and treatment of CVDs.

One of the primary applications of 3D cardiac constructs is to mimic human diseases for the study of pathogenic mechanisms and drug screening. Their ability to allow real-time measurement of pathological phenotypes in vitro makes them particularly useful for evaluating drug treatment effects. Additionally, the small size of 3D cardiac constructs is advantageous for constructing high-throughput drug screening platforms, thereby reducing the reliance on experimental animals. Consequently, 3D cardiac constructs have emerged as valuable preclinical platforms for evaluating drug efficacy in drug discovery.

3D cardiac constructs are classified into various types, each with distinct characteristics. A deeper understanding of these models is essential for selecting or optimizing them for specific applications. This article provides a brief overview of the latest advancements in 3D cardiac constructs research and discusses their application in disease modeling and drug screening. These insights aim to offer theoretical guidance for disease modeling and drug discovery based on 3D cardiac constructs.

3D cardiac constructs technology holds immense promise for revolutionizing cardiovascular research and regenerative medicine, yet substantial challenges persist that limit its clinical translation. Despite advancements in mimicking human heart tissue structure and function, key hurdles remain, including the difficulty in achieving mature, electromechanically coupled 3D constructs that fully replicate adult cardiomyocyte physiology. Additionally, large-scale fabrication for high-throughput applications and the need for improved vascularization to enable nutrient delivery in larger constructs pose ongoing limitations. These barriers must be addressed to improve the potential of this technology for applications including disease modeling and drug screening.

## The 3D Cardiac Constructs

The field of 3D cardiac constructs technology is undergoing rapid development, and so is the CVD research area. The discovery of hPSCs, including human induced pluripotent stem cells (iPSCs) and human embryonic stem cells (hESCs), has made it possible to explore human cardiomyogenesis and the underlying causes of both congenital and late-onset cardiac disorders [3,4]. The challenge of limited availability of patient-derived primary cells has been addressed by iPSC technology, enabling the advancement of high-fidelity disease models. Furthermore, various complex and monogenic cardiac pathologies have been investigated in vitro with iPSC-derived cardiomyocytes (iPSC-CMs) and genome editing techniques, providing new insights into disease mechanisms [5]. Additionally, human iPSCs-CMs have become crucial tools in both drug development and repurposing [6].

**Fig. 1.**
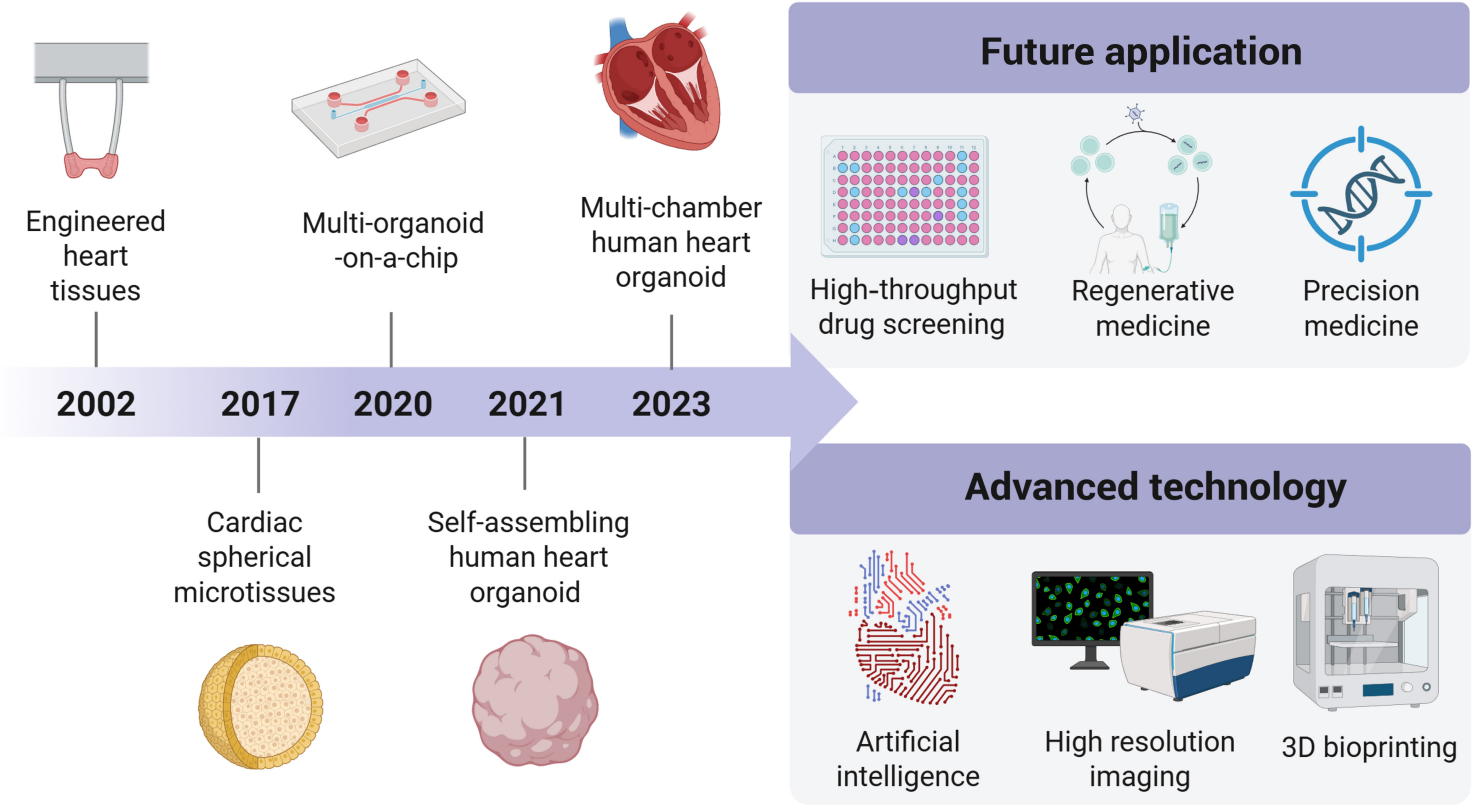
Graphical timeline and future direction of 3D cardiac constructs. This figure illustrates the roadmap summarizing key milestones in 3D cardiac constructs development and future directions.

Following the establishment of cardiomyocyte differentiation systems induced by hPSCs, multiple in vitro 3D cardiac constructs have been developed, each with unique features and capabilities (Fig. 1). Engineered heart tissue (EHT) was first generated from neonatal rat cardiomyocytes mixed with collagen I in 2002 [7]. Human EHT was then generated from hESCs in 2009 using activin A and BMP4 in a suspension culture system [8]. EHT exhibited spontaneous contraction and electrophysiological properties, making it suitable for drug testing and transplantation. The concept of heart-on-chip emerged around 2014 and typically refers to a small chip containing a network of microchannels and cardiac cells [9]. This system enables real-time measurement of cardiac contractility and electrophysiological functions, facilitating high-throughput drug testing [10,11]. With the advancement of technology, the so-called body-on-chip integrating various organoids (including cardiac organoid) was developed around 2020 [12]. Scaffold-free cardiac spherical microtissues, composed of cardiomyocytes and other cell types, were developed around 2017. These microtissues, commonly self-organized in suspension culture, are easy to construct and small in size, making them suitable for drug screening [13–15]. The self-assembling human heart organoids (hHOs) (so-called cardioid) are self-organized structure with chamber, derived from human PSCs that mimic early heart development, which appeared in 2021 [16,17], and then multi-chamber cardioids were established in 2023 [18]. These 3D models replicate key features of embryonic hearts, including cavity formation and multi-lineage cell organization. On anti-adhesion surfaces, hPSCs typically self-assemble into aggregates that differentiate into cardiac cells within organoids [16,19]. This system is particularly well-suited for studying cardiogenesis and congenital heart disease.

**Fig. 2.**
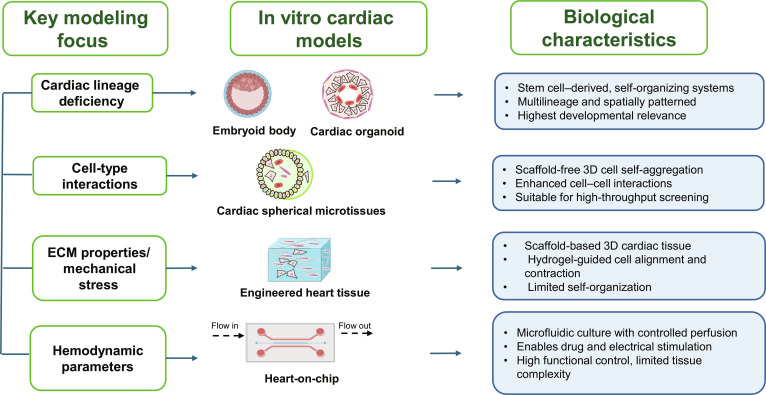
Various in vitro 3D cardiac constructs and key modeling focus. This figure illustrates various in vitro 3D cardiac constructs and their major features, highlighting advancements in cardiac tissue engineering and pathological/physiological research.

The distinctions among human EHT, heart-on-chip, scaffold-free cardiac spherical microtissues, and cardioids lie in their structural complexity, functional features, and underlying mechanisms (Fig. 2 and Table 1). EHTs are traditionally fabricated using scaffolds or hydrogel to support cardiomyocyte growth, enabling 3D organization and electromechanical coupling, which better recapitulates native heart tissue architecture [20]. In contrast, heart-on-chip systems integrate microfluidic channels with embedded cardiomyocytes on flexible substrates, allowing precise control of mechanical and biochemical cues, such as fluid shear stress and oxygen gradients, to simulate dynamic physiological environments and enhance relevance for drug testing and disease modeling [9,11]. Scaffold-free cardiac spherical microtissues, however, self-assemble into spheroidal structures through cell–cell adhesion and extracellular matrix secretion, eliminating scaffold dependence and promoting cellular communication via gap junctions and paracrine signaling, which improves tissue maturation [14,21]. Cardioids, as advanced microphysiological systems, specify human stem cell into heart-like cavity structures in engineered microenvironments to model cardiac development and disease with high fidelity, emphasizing organotypic complexity and biochemical responsiveness [16]. At the mechanism level, EHTs rely on the property of scaffold and matrix for tissue integration, while heart-on-chip systems use microfluidic dynamics to mimic circulation. Scaffold-free microtissues exploit stem cell aggregation capacity, and cardioids depend on stem cell differentiation signals. Collectively, these platforms vary in scalability, biological relevance, and application focus, with EHTs and cardioids excelling in structural realism, heart-on-chip in dynamic modeling, and scaffold-free microtissues in simplicity and adaptability. For experiment design, cardioids offer high physiological fidelity by mimicking human heart development and disease progression, making them ideal for studying complex cardiac pathologies. In contrast, microfluidic heart-on-chips are superior in throughput and scalability, enabling rapid screening of drug candidates or environmental stressors. Cardioids prioritize accuracy at the expense of throughput and cost efficiency, while heart-on-chips sacrifice some organ-level complexity for efficiency. By comparing these models, including cardioids’ strength in disease modeling versus heart-on-chips’ advantages in high-throughput assays, researchers should choose their experimental platform with their priorities, whether they seek mechanistic insights or scalable solutions.

**Table 1.** Comparative overview of 3D cardiac constructs

Model type	Cell source requirements	Structural complexity	Functional readouts	Scalability for HTS	Key advantages	Primary limitations	Reference
EHT	IPSC-derived cardiomyocytes co-cultured with cardiac fibroblasts, engineered into 3D force-generating tissues that undergo progressive maturation.	Anisotropic/elongated cardiomyocytes, 3D mechanically suspended tissue. No epicardium, endocardium, or vasculature.	Exceptional electrophysiological, metabolic, and mechanical maturation markers, contractile force and drug responsiveness.	Low-moderate throughput	Robust cardiomyocyte maturation. Good drug sensitivity, physiologically relevant mechanical loading.	Technically demanding setup; limited biological complexity; no epicardium, endocardium, or vasculature.	[28]
Heart-on-a-chip	IPSC-derived cardiomyocytes; fibroblasts; vascular endothelial cells; 3D co-culture system.	3D heart-on-a-chip model; stacked myocardial tissue and vascular endothelial cell layers. Mimics anatomical structure of cardiac tissue and high structural complexity.	Contractile function, vascular permeability, CD31 expression, F-actin remodeling, endothelial alignment under shear stress.	Moderate, requires perfusion systems	Physiologically relevant cardiac–vascular interactions; ability to assess vascular permeability.	Lacks immune cells and vascular smooth muscle cells; inflammatory responses not yet modeled and limited range of tested drugs.	[133]
Scaffold-free microtissues	IPSC-derived cardiomyocytes with automatically self-arising populations of epicardial, fibroblast, and endocardial cells, formed without separate lineage differentiation.	3D self-organized, scaffold-free microtissues with multicellular organization and ultrastructural maturation of sarcomeres and mitochondria.	Long-term (>100 days) spontaneous heart contractions; transcriptional and metabolic maturation; dose- and time-dependent responses to cardiotoxic drugs; functional responses to cardioactive drugs.	Moderate; simple static culture without external scaffolds supports long-term maintenance	Scaffold-free self-organization, extended culture duration, improved survival compared to 2D cultures.	Immature (fetal-like) cardiac phenotype; lack of external mechanical or perfusion-based stimulation.	[134]
Cardioids	Cardiac mesoderm with stepwise incorporation of 1, 2, or 3 main cardiac lineages; generated without exogenous ECM and without non-cardiac tissues.	Self-organizing organoids forming chamber-like cavities; architectural complexity is tunable depending on the number of incorporated cardiac lineages.	Morphogenetic patterning, chamber-like cavity formation, and spatial–temporal dissection of signaling factor functions.	High; high-throughput approach with controlled signaling increases reproducibility and statistical power	Reduced variability compared to other organoids; modular and simplified system; controllable self-organization; complexity tailored to the biological question.	Recapitulates only a subset of intrinsic developmental mechanisms and differs from embryogenesis.	[16]

EHT, engineered heart tissue; ECM, extracellular matrix; HTS, high-throughput sequencing

### Genetic cardiac 3D disease models generated by patient iPSCs with specific gene mutations

A pivotal strength of iPSCs in disease modeling is that they are genetically matched to the donor, making them particularly suited for modeling diseases with a defined genetic cause. Since iPSCs are reprogrammed from adult cells into a pluripotent state, many epigenetic changes induced by environmental influences are erased. Consequently, for non-genetic disease modeling, external stimuli must be re-applied [22]*.* Several genetic cardiac 3D disease models have been established employing iPSCs derived from patients carrying mutations associated with CVDs (Table 2). In Table 2, we compile various genetic cardiac 3D disease models along with their corresponding genetic alterations, pathological manifestations, and testing methodologies. Cardioids derived from hPSCs have been employed to investigate genetic cardiac defects (such as conditions leading to arrhythmias and reduced contractility) using advanced techniques including gene expression profiling and electrophysiological testing [16,19]. iPSC lines carrying mutations in C10orf71 have been used to generate organoids, while gene editing of dilated cardiomyopathy (DCM) models with contractility defects has facilitated the evaluation of different stages of functional and pharmacological responses [23]. 3D cardiac constructs have also been used to study arrhythmogenic cardiomyopathy (ACM), where they exhibited loss of contractile function, validating their utility for drug screening and gene expression analysis [24]. Hypertrophic cardiomyopathy (HCM) has been modeled using ACTN2-mutant hiPSC-derived cardiomyocytes, which successfully replicated key pathological features such as hypertrophy, hypercontractility, and myofibrillar disarray. Subsequent functional assays with L-type calcium channel blockers such as diltiazem demonstrated clinical benefit by enhancing contraction and relaxation [25]. In summary, 3D cardiac constructs are particularly well-suited for simulating the phenotypic manifestations of cardiac diseases caused by genetic mutations and underpin the clinical translation of drug discovery.

**Table 2.** Genetic 3D cardiac constructs

Type	Pathogenesis	Pathological phenotype	Testing method	Key limitations and translational relevance	Reference
Cardioids	HAND1 loss of function caused heart defects	Structural defects (e.g., malformed myocardium), impaired contractility	Imaging, electrophysiological assays, mechanical force measurements, gene expression profiling	Limited lineages/cell types; early stage modeling only. Protentional for drug discovery and regenerative research.	[16]
iPSC-derived cardiac organoid	Mutation in C10orf71 causing dilated cardiomyopathy (DCM)	Impaired cardiomyocyte contractility, preserved sarcomere structure	Functional assays of organoids, pharmacological testing (e.g., omecamtiv mecarbil)	Small sample; focused on DCM and LOF variants; mechanism not fully confirmed. Useful for patient-specific modeling and functional studies.	[23]
iPSC-derived cardiac organoid	NKX2.5 loss of function caused cardiac malformations	Decreased cardiomyocyte adhesion, hypertrophy	Gene expression analysis	Early-stage modeling only; incomplete patterning; protocol-sensitive. Useful for high-throughput disease modeling and drug discovery.	[19]
hiPSC-derived cardiomyocytes and engineered heart tissues	Mutation in ACTN2(c.740>T) causing hypertrophic cardiomyopathy (HCM)	Hypertrophy, myofibrillar disarray, hypercontractility, impaired relaxation, prolonged action protentional, and enhanced L-type Ca^2+^ current	Functional assays for contraction and relaxation, action potential measurement, and drug testing with L-type Ca^2+^ channel blocker diltiazem	Control variability and incomplete mechanistic insight; long-term effects unknown. Directly guides patient therapy and personalized drug testing.	[25]
iPSC-derived cardiomyocytes	Mutation in TNNT2 causing dilated cardiomyopathy (DCM)	Contractile and metabolic dysfunction, mitochondrial respiration defects, reduced ATP levels	Phenotypic screening with small molecule kinase inhibitors (SMKIs) to identify therapeutic targets; metabolic and contractile assays	Fetal-like phenotype; molecular mechanisms sometimes unclear. Useful for drug discovery and studying human CM function.	[90]
Engineered heart tissue (EHT) from hiPSC-derived cardiomyocytes	Knockout of the de novo DNA methyltransferase DNMT3A, impacting DNA methylation in cardiomyocyte physiology and disease	Impaired mitochondrial metabolism and altered glucose metabolism, hypertrophic signaling	DNA methylation, gene expression, hypertrophic signaling induced by endothelin-1 and phenylephrine; assessment of contractility, morphology	Immature cardiomyocytes; rescue limited to hypertrophic conditions. Useful for studying metabolism, hypertrophy, and functional rescue in human EHTs.	[135]
Bioengineered tissue from hiPSC-derived cardiomyocytes	PKP2^R413X^ truncating variant causing arrhythmogenic cardiomyopathy (ACM)	Impaired myofibrillogenesis, delayed mechanical coupling, and reduced calcium wave velocity in engineered tissues	Functional assays for mechanical coupling, cytoskeletal organization, and calcium wave propagation; Wnt/β-catenin modulation with SB216763	Immature hiPSC-CM phenotype; variant- and pathway-specific effects; safety concerns with long-term signaling modulation. Human-relevant ACM modeling and testing of genotype-specific therapeutic strategies.	[136]
hiPSC-derived cardiomyocytes and organoids	Loss of HOPX gene function impacting genomic regulation of heart development	Impaired cardiac gene regulation, disrupted enhancer networks, and altered cardiomyocyte function	Genetic loss-of-function assays, gene expression profiling, and functional studies in cell, organoid, and zebrafish regeneration models	Immature hiPSC/organoid models; lacks mammalian in vivo validation; highlights HOPX as a potential target in cardiac development and regeneration	[137]
3D cardiac microtissues with human iPSC-derived stromal cells	ACM caused by the heterozygous c.2013delC PKP2 mutation in cardiac fibroblast	Abnormal contraction	Mechanical contraction, Ca^2+^ handling, mitochondrial respiration capacity	Fetal-like CMs; need extra cues for full maturation; lacks systemic context. Low-cost, reproducible, multicellular human model for disease modeling and drug testing; patient-specific.	[24]

ACM, arrhythmogenic cardiomyopathy; DCM, dilated cardiomyopathy; EHT, engineered heart tissue; HCM, hypertrophic cardiomyopathy; LOF, loss of function; SMKIs, small molecule kinase inhibitors

### Non-genetic CVD 3D models affected by environmental factors

Beyond genetic determinants, non-genetic variables including chronic inflammation, metabolic alterations, and environmental stressors also play a crucial role in disease progression. Currently, many non-genetic CVDs have been successfully recapitulated by organoid models (Table 3), which simulate how these environmental factors affect mixed cellular populations, including cardiac, endothelial, and vascular. Functional testing, transcriptomics, and immunostaining have demonstrated that cardiac organoids exposed to metabolic stress and environmental pollutants exhibit phenotypes associated with oxidative stress and inflammation, leading to structural defects and reduced contractility (Table 3). Vascular organoids can model endothelial dysfunction, vascular stress, and reduced vessel branching, which might be influenced by environmental toxins and chronic inflammation [26]. These models are evaluated using sophisticated techniques such as gene expression analysis, elasticity measurements, and live imaging [26]. With the help of tissue imaging and functional testing, heart assembloids containing tissue-resident macrophages replicate normal immune–cardiac interactions, allowing the study of immune-mediated heart disease, inflammation, and altered contractility [27]. In summary, cardiac 3D models provide a valuable platform for investigating the pathogenic mechanism of CVDs influenced by environmental factors and offer key insights for drug discovery.

**Table 3.** Non-genetic 3D cardiac constructs

Type	Pathogenesis	Pathological phenotype	Testing method	Key limitations and translational relevance	Reference
Cardiac organoids	Environmental toxins and metabolic stress trigger oxidative stress and inflammation	Structural abnormalities, impaired contractility	Immunostaining, functional assays, transcriptomics	Not fully adult-like, but a scalable human 3D platform to study cardiomyocyte maturation mechanisms and enable drug screening targeting metabolism and cardiac regeneration.	[138]
Vascular organoids	Chronic inflammation, environmental toxins, vascular, stress	Vascular stiffening, impaired endothelial function, reduced vessel branching, increased permeability	Live imaging, elasticity measurements, immunostaining, gene expression analysis, 3D culture systems, fluid shear stress experiments	Lacks full tissue complexity; still simplified compared to in vivo vasculature. Multicellular, patient-specific 3D model; better mimics native vascular responses; useful for drug testing and organoid integration.	[26]
Heart assembloids with autologous tissue-resident macrophages	Recreates physiological immuno-cardiac interactions, immune-mediated heart disease	Inflammation, immune response, altered contractility	Functional assays, tissue imaging	Still in vitro; lacks full vascularization, mechanical forces, and systemic signals; human-specific, multicellular model with tissue-resident macrophages for studying development, arrhythmias, inflammation, and drug testing.	[27]
Human cardioid platform	Impact of drugs, and environmental factors on heart compartment development	Compartment-specific defects in the ventricles, atria, outflow tract, and atrioventricular canal; disrupted signal and contraction propagation	Gene expression profiling, functional assays, and teratogen/drug testing	Limited to early heart development; or overall growth/maturation; mainly validated for early-stage mutations and teratogen studies.	[18]

2D, 2-dimensional; 3D, 3-dimensional

### Impact of different cell sources on constructing in vitro cardiac models

The selection of cell types derived from PSCs critically shapes the functionality and disease relevance of cardiac organoids. Cardiomyocytes (CMs) form the core contractile units, enabling organoids to mimic heart rhythm and response to stimuli [7], but their maturation level often falls short of adult cardiomyocytes [28], limiting physiological fidelity. Endothelial cells are essential for vascularization [29], facilitating nutrient delivery and waste removal. Cardiac fibroblasts (CFs) provide structural support and influence extracellular matrix remodeling [24], but their overrepresentation may shift organoid behavior toward fibrosis rather than healthy cardiac dynamics. Meanwhile, immune cells like macrophages, when incorporated, enhance inflammation modeling and response to injury, though their presence introduces variability due to complex cell–cell interactions [27]. Each cell type offers distinct advantages: CMs for contractility, endothelial cells for perfusion, CFs for tissue integrity, and macrophages for immune modulation. Optimizing ratios and differentiation protocols is thus pivotal to balance organoid realism with experimental reproducibility, ensuring they serve as robust models for cardiac development, disease, and therapy testing.

## 3D Cardiac Constructs Readouts and Testing Methods

**Fig. 3.**
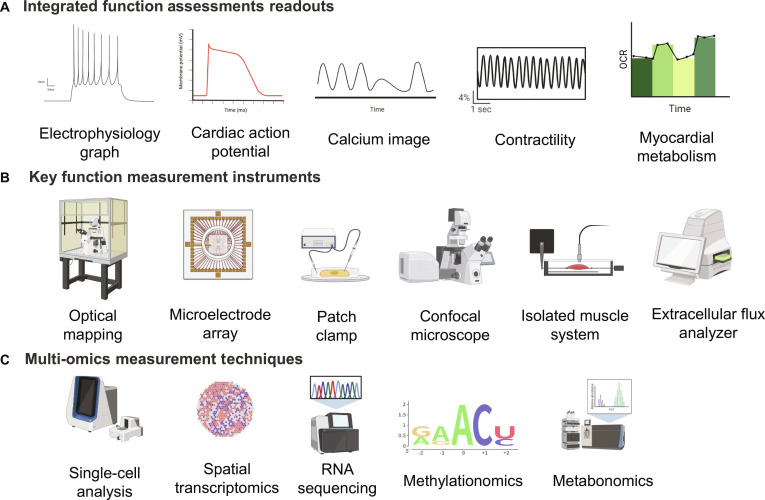
Readouts and the testing methods. (A) Readout of cardiac functions in 3D cardiac models, including electrophysiology, contraction, and metabolism of myocardium. OCR, oxygen consumption rate. (B) Testing methods and equipment in 3D cardiac models. (C) Applicable multi-omics technologies in 3D models.

Ongoing advancements in hPSC-derived 3D cardiac constructs rely on novel technologies or the adaptation of existing technologies to further investigate physiological and pathological mechanisms [30]. Various quantitative and qualitative methods are used to assess their morphology and function (Fig. 3). These methods are essential for evaluating the accuracy and sensitivity of the 3D models and for collecting meaningful experimental readouts.

### Electrophysiological assessments

Electrophysiology is one of the key parameters in assessing heart function (Fig. 3). Commonly used methods are discussed in the following sections.

#### Multielectrode array recording

The development of multielectrode array (MEA) in cardiology has enabled scientists to evaluate the electrophysiological properties of cardiac organoids and cardiomyocytes, providing a non-invasive method for monitoring heart rhythm and conduction. MEA devices consist of arrays of microelectrodes embedded in a substrate, allowing for the recording of extracellular potentials from cells [31]. This technology provides a high-resolution view of network dynamics, tissue connectivity, and cellular behavior. MEA has revolutionized the understanding of cardiac tissues and neural networks, where electrical activity underpins the functionality of both physiological function and disease pathogenesis [31]. It serves as a crucial platform for disease modeling and drug screening, enabling researchers to measure electrophysiological changes in cardiac tissues in response to pharmacological compounds, genetic modifications, and environmental stressors. This approach not only enhances our understanding of heart conditions but also holds potential for personalized medicine and the design of novel therapeutic approaches for patients with arrhythmias and other heart disorders [30,31]. However, the integration of sensors within organoids may disrupt their 3D structure and alter normal physiology [32]. Therefore, it is essential to develop MEA devices capable of assessing electrophysiological properties in intact 3D organoids [33,34].

#### Patch-clamp technique and optical mapping

The patch-clamp technique facilitates precise evaluation of cellular membrane potential and provides high-resolution analysis of cardiomyocyte electrophysiology [35–37]. It is a widely used electrophysiological method for assessing the electrical characteristics of individual cells, particularly those in cardiac tissues. This technique involves the insertion of a fine-tipped, high-resistance electrode into a cell, allowing for the accurate recording of cellular membrane electrical activity [31]. Optical mapping is another approach that utilizes a voltage-sensitive dye, which changes intensity in response to membrane potential alterations. A previous study demonstrated the electrophysiological characteristics of cardiac microtissues using optical mapping [38], showing that cardiac microtissues serve as effective models for assessing the proarrhythmic risk of drugs. A fundamental strength of optical mapping is its ability to record action potentials in intact organoids without disrupting their native 3D structure. However, dye-induced cytotoxicity and photobleaching limit its applicability for long-term culture and repeated recordings.

### Calcium signaling

In addition to patch-clamp and MEA, voltage-sensitive dye-based calcium imaging is a prominent technique for assessing organoid functionality, particularly when maintaining structural integrity is essential for readout acquisition [19,39,40]. Calcium imaging enables the monitoring of intercellular calcium concentration changes, facilitating research on excitation–contraction coupling [41,42]. The processes of calcium uptake, release, and buffering dynamics are essential for coordinating cardiomyocyte contraction, underscoring the importance of replicating these mechanisms in organoids [41]. This field has been further advanced by state-of-the-art calcium imaging techniques, which provide powerful tools for analyzing calcium handling in cardiac tissues that have undergone genetic or pharmacological modifications [42]. In recent studies, a customized spectral-domain optical coherence tomography system was employed to enable fast, label-free, and non-invasive 3D imaging of hHOs [43,44]. Calcium imaging was concurrently utilized to visualize the dynamic beating patterns of hHOs. Together, optical coherence tomography and calcium imaging form a synergistic multimodal platform that correlates structural morphogenesis with physiological function, offering a powerful in vitro model for studying early heart development and disease mechanisms. However, dye-induced cytotoxicity and photobleaching limit the long-term use of voltage-sensitive dyes in culture [45]. To address these challenges, genetic calcium reporters have emerged as effective alternatives for studying cardiac calcium signaling [45,46].

### Contractility measurements

By combining optimized algorithms with genetic calcium reporters, a method utilizing standard video microscopy has been developed to simultaneously analyze the coupling of calcium flux and contractility in human iPSC-CM [47]. The readouts include contraction magnitude, beating rate, and beating velocity, which have been widely used in conventional cardiomyocyte research. The open-source software used in this study was validated through drug response analysis and can be modified and applied to engineered microtissues. However, in contrast to 2D monolayer cultures, cardiac organoids on ultra-low attachment surfaces can experience positional and orientation shifts as a result of hypercontractile activity. This phenomenon warrants inclusion in the analytical framework to avoid miscalculations of contraction magnitude [48].

### Metabolism

Key factors such as energy output, mitochondrial function, and substrate utilization in organoid models can be analyzed using various readouts and testing techniques. One of the primary functions of cardiomyocyte respiration is to generate adenosine triphosphate (ATP) required for the myocardium contraction from energy-providing substrates, including fatty acids, lactate, and glucose. Although multiple pathways contribute to energy production, mitochondrial oxidative phosphorylation accounts for approximately 90% of total energy generation [49]. Recent studies have demonstrated that cardiac organoids effectively model key metabolic processes, such as changes in mitochondrial activity and substrate utilization under both physiological and pathological conditions [17,50]. Using cutting-edge techniques such as metabolomics and Seahorse XF analysis, researchers have identified novel therapeutic targets and evaluated drug-induced cardiotoxicity by gaining crucial insights into energy metabolism and disease phenotypes. The Seahorse Extracellular Flux Analyzer facilitates real-time, high-precision quantification of oxygen consumption rate (OCR) and extracellular acidification rate (ECAR) in living cells [49,51]. OCR reflects mitochondrial respiration, while ECAR corresponds to proton excretion via glycolysis. These parameters allow researchers to assess mitochondrial oxidative phosphorylation and glycolytic activity, providing a comprehensive view of the metabolic state of cardiac organoids [52]. Furthermore, real-time cell respiration assays dynamically track dissolved oxygen levels and proton flux in extracellular media [51]. Variations in OCR and ECAR are directly linked to ATP turnover, reflecting ATP synthesis and consumption rates, which are critical for understanding cardiac energy metabolism [53].

### Genetic profiling

In addition to metabolic profiling, which provides crucial insights into the bioenergetic state and functional condition of cardiac organoids, a complementary analysis of gene expression is also necessary to understand the underlying molecular mechanisms. Multi-scale analyses of the genome, transcriptome, proteome, and metabolome are fundamental for identifying pathological biomarkers and gaining insights into the mechanisms underlying the pathogenesis of CVDs [54]. Gene expression studies in cardioids have identified key pathways associated with developmental heart chamber defects, including mesodermal WNT and BMP signaling and the HAND1 transcription factor [16]. Additionally, time-course RNA sequencing analysis during differentiation has revealed the production of pivotal morphogens, growth factors, and receptors at various cardiac developmental stages [17]. In terms of developmental mechanisms, a study constructed single-cell molecular maps and spatial maps during cardiac development using single-cell RNA sequencing and multiplex error-robust fluorescence in situ hybridization technology, revealing the regional specialization of cell subpopulations and the formation mechanism of cell community structures [55]. This study establishes a theoretical foundation for utilizing cardiac organoids to model heart development. These findings highlight how molecular insights into cardiovascular development and disease can be obtained from cardiac organoids, providing valuable models for studying cardiac pathophysiology and potential therapeutic targets.

## Biomaterial Enhanced 3D Cardiac Constructs

To conduct these functional measurements in clinically relevant models, the integration of advanced biomaterials with tailored mechanical and biochemical cues is indispensable. The development of novel materials is continuously driving advancements in 3D cardiac constructs functional enhancement. Current mainstream fabrication technologies primarily include 3 approaches: (a) suspension culture methods utilizing rotating bioreactor systems, (b) Matrigel embedding techniques supported by hydrogels or decellularized matrices, and (c) 3D bioprinting for precise spatial control of cell deposition [56]. The emergence of advanced biomaterials, particularly photopolymerizable hydrogel (the photo-initiator absorbs light to generate free radicals that cross-link monomers into a 3D network) and conductive nanofibers, has overcome critical challenges in structural precision and electrical signal transmission that previously restricted conventional 3D culture systems. This technological breakthrough enables the engineering of complex tissue models, including vascularized 3D cardiac constructs with enhanced physiological relevance. Recent advancements have shown that integrating organoids with microfluidic chip technology enables the creation of functional “human-on-a-chip” systems capable of simulating multi-organ interactions [57]. This breakthrough provides novel opportunities for investigating cross-organ pharmacological effects in vitro.

### Conductive biomaterials in 3D cardiac constructs

Recent breakthroughs in cardiac tissue engineering have witnessed expanding applications of conductive biomaterials. These functional materials serve dual purposes: they can be utilized as substrates for cardiomyocyte culture in vitro or engineered into conductive cardiac patches for implantation into MI regions [58–60]. Experimental studies have demonstrated that culturing H9C2 rat cardiomyocytes on graphene-incorporated conductive composite membranes with charged surface properties markedly enhances cellular adhesion and promotes proliferation rates [61]. These findings substantiate the suitability of such conductive biomaterials for cardiac tissue engineering applications. A research team has successfully developed conductive electroactive fiber scaffolds with an elastic modulus matching that of natural myocardium [62]. When cultured on these scaffolds, H9C2 cells demonstrate enhanced adhesion, migration, and proliferation capabilities. The scaffolds effectively guide cardiomyocyte alignment along the fiber orientation while simultaneously promoting angiogenesis. Notably, these scaffolds exhibit excellent biocompatibility in rat models [62]. By precisely adjusting the fiber architecture and scaffold composition, advanced modeling techniques mimic the intricate microstructure of cardiac tissue with precision. In another example, when culturing murine embryonic stem cell-derived cardiomyocytes (ESC-CMs) within engineered matrices, the incorporation of graphene-based biohybrid matrices markedly enhances cellular metabolic activity and facilitates sarcomere development, demonstrating superior matrix compatibility [63]. Furthermore, the application of electrical stimulation through these conductive biohybrid matrices yields additional benefits, driving substantial improvements in ESC-CM organization and physiological maturation [63].

Moreover, the development of “nano-conductive” cardiac organoids, a construct engineered through the integration of conductive silicon nanowires (e-SiNWs) with hPSC-derived CMs (hPSC-CMs), demonstrates dual functional enhancements: improved electrical conduction capacity and enhanced intercellular electrical synchronization [64]. After transplantation into myocardial infarct models, these constructs exhibit measurable therapeutic benefits, including reduced fibrotic area and improved ventricular ejection fraction. The e-SiNWs effectively mimic native cardiomyocyte electrical conduction pathways, thereby establishing functional electrical coupling between the transplanted organoids and host myocardium, ultimately enhancing MI treatment outcomes [64]. Previous research has demonstrated the development of a self-powered triboelectric nanogenerator patch designed to generate electrical stimulation through cardiac contraction simulation [65]. This innovative device effectively delivers therapeutic stimulation to infarcted myocardial regions, thereby enhancing electrical signal propagation between healthy and damaged cardiac tissues. Furthermore, the supplemental electrical stimulation provided by the patch accelerates cardiomyocyte maturation and functional development. In porcine MI models, implementation of this generator patch yielded 2 important benefits: (a) increased electrical conduction velocity within the infarct zone, and (b) capability for wireless electrocardiographic signal monitoring, achieving an integrated approach combining both treatment and diagnostic functions [65]. A recent study used biopolymers combined with conductive materials and 3D printing technology to simulate the layered structure of natural cardiac tissue, enabling the printing of multi-layered structures containing cardiomyocytes, endothelial cells, and stromal cells [66]. The incorporated conductive components serve multiple critical functions: (a) facilitating intercellular electrical signal propagation, (b) promoting synchronous electromechanical coupling, (c) enhancing functional maturation of cardiomyocytes, and (d) maintaining rhythmic contractile activity. Collectively, these features greatly improve the electrophysiological performance and functional integration of the engineered cardiac tissues [66].

### Hydrogel materials in 3D cardiac constructs

In cardiac tissue engineering, hydrogel materials have gained extensive research attention owing to their dual functional advantages of injectability and transplantability. These versatile biomaterials can be either directly injected into myocardial tissue or fabricated into cardiac patches via 3D printing technology for precise implantation into infarcted regions [67]. Notably, the application of 3D printing technology with biocompatible hydrogel materials permits precise spatial control of diverse cell types within organoids, effectively mimicking the cellular architecture of the developing heart [68]. This technological advancement enables cardiac organoids to more faithfully replicate developmental processes, providing critical insights into both the pathogenesis of congenital heart defects and the pharmacological responses of the embryonic heart [68]. Notably, hydrogel-based systems incorporating bioactive factors demonstrate synergistic therapeutic effects. A representative example involves the injection of conductive hydrogels loaded with human mesenchymal stem cell-derived exosomes (hMSC-exosomes) into MI models [69]. This combined approach provides dual therapeutic benefits: (a) the conductive matrix facilitates sustained exosome release while restoring cardiac electrical conduction, and (b) the exosomes exert multifaceted regenerative effects including angiogenesis promotion, apoptosis inhibition, and arrhythmia reduction. Recent advances have substantially broadened the therapeutic scope of this approach. Experimental studies have demonstrated that a pH-responsive multifunctional conductive hydrogel (OHA/Col-CDH/MWCNT) loaded with metformin and exosomes synergistically regulates oxidative stress, electrical conduction, and angiogenesis through an intelligent release system [70]. This combinatorial strategy has been shown to markedly enhance myocardial regeneration outcomes in preclinical models [70]. In the field of bionic structural engineering, researchers have developed fiber-reinforced hydrogel inks for 3D bioprinting of cardiac-mimetic scaffolds [71]. These advanced constructs precisely recapitulate both intracellular and extracellular cardiac tissue architectures. The embedded fibrous network not only promotes anisotropic cell alignment but also faithfully reproduces the heart’s spatiotemporal excitation–contraction coupling dynamics at the organ scale [71]. Recently, researchers have successfully combined hiPSC-CMs with collagen hydrogels to create functional “cardiac patches” [72]. In rhesus monkey models, these patches restored cardiac function by thickening ventricular walls and improving ejection fractions. Human trials confirmed sustained functional benefits in heart failure patients, offering a bridge to transplantation and a novel regenerative approach.

The improvement of the conductive properties of hydrogels is of vital importance for enhancing their functionality in 3D tissue construction. A groundbreaking conductive hydrogel with mechanical and electrical properties mimicking native myocardial tissue [73] overcomes biocompatibility limitations of conventional hydrogels. This material offers 5 key benefits: (a) minimal inflammatory response, (b) enhanced cardiomyocyte maturation, (c) guided sarcomere assembly, (d) infarct size stabilization, and (e) extended stem cell therapy viability. These attributes position it as a superior scaffold for bioengineered cardiac organoids. Conductive hydrogel patches offer unique advantages for cardiac repair by precisely replicating the electrophysiological properties of myocardial tissue [74]. Their matched conductivity restores synchronized electrical activity in infarcted regions, while preclinical studies demonstrate multiple therapeutic benefits: they enhance electrical signal conduction, accelerate impulse propagation at lesion sites, promote angiogenesis, and reduce cardiomyocyte apoptosis [74]. These synergistic effects highlight their potential in improving heart function post-infarction.

### Electromechanically integrated materials in 3D cardiac constructs

Biomedical nanofabrication and integrated sensing technologies enhance cardiac organoid maturation by precisely simulating the biomechanical microenvironment, including tensile forces and fluid shear stress. Recent studies show that multimodal, implantable sensors synchronize with organoid growth, creating an “intra-organoid interface” for long-term electrophysiological monitoring and stimulation [75]. A key advancement is the dual functionality of embedded electrodes: they not only record electrophysiological signals but also actively regulate development by influencing cardiomyocyte maturation. Specifically, electrical pulses improve sarcomere alignment and calcium transient synchronization. Additionally, these sensors enable real-time tracking of functional parameters (e.g., contraction frequency and pressure dynamics), overcoming limitations of conventional optical methods that lack comprehensive morphological and functional data [75]. Micropore arrays provide geometric constraints that drive cellular self-organization, offering a precisely controlled microenvironment for cardiac organoid development [32]. Co-culturing hiPSC-CMs with rat cardiac endothelial cells within these micropores promotes cell aggregation and spontaneous contraction. The resulting anisotropic stress gradients, combined with vascular network formation, facilitate the assembly of multi-chamber structures, including pacemaker cells, fibroblasts, and cardiac layers, that mimic native heart architecture and improve cardiomyocyte maturation [32]. In addition to improving cardiac function, nano-fabrication and integrated sensing technologies also present an innovative framework for drug screening in CVDs. The 3D-printed flexible nano-silver electrode array (FlexNEA) can capture intracellular signals through electroporation, synchronously recording contraction stress and action potential conduction velocity [76]. This array enables high-fidelity recording of cardiomyocyte action potential dynamics under pharmacological modulation by specific ion channel blockers, showcasing robust drug screening utility. It provides precise, quantitative assessment of ion channel drug effects, thereby establishing a novel high-precision platform for evaluating drug-induced arrhythmogenic risk [76].

### Biomaterial in vascularized 3D cardiac constructs

Furthermore, for the construction of vascularized cardiac organoids, co-culturing endothelial cells with biocompatible matrices enables the development of perfusable microvascular networks. This approach better recapitulates in vivo blood perfusion and nutrient delivery, consequently improving the physiological fidelity of organoid systems [77,78]. It is worth noting that cardiac organoids with spatial structures and vascular networks can be constructed by optimizing culture formulas and using specific combinations of growth factors, overcoming the problems of limited organoid size and maturity due to the lack of a vascular system [79]. Such vascularized cardiac organoids offer superior capabilities in recapitulating cardiac physiological functions and investigating drug effects on cardiac development. From a materials innovation perspective, recent studies have demonstrated a novel strategy: modulating matrix viscoelasticity to guide hPSC-derived vascular organoids toward forming arteriole-like structures [77]. In this technology, dynamic hydrogels facilitate outward vascular expansion, yielding complex networks with enlarged luminal diameters. This approach greatly enhances arteriole reorganization in infarcted hearts and improves post-transplant functional recovery, offering a transformative solution for vascularized cardiac organoid engineering and functional maturation.

Currently, multiple technical approaches have emerged for constructing vascularized cardiac organoids. Conducting detailed comparative analyses of different vascularization strategies holds substantial practical guidance value: co-culture represents a commonly employed method. This involves co-culturing endothelial cells with biomaterials and cardiac lineage cells, mimicking in vivo paracrine effects and the microenvironment of intercellular interactions to achieve spontaneous vascular network formation. This approach more authentically simulates the organ’s blood flow and nutrient supply [77]. The process is relatively straightforward, yet suffers from poor controllability of vascular distribution and insufficient network regularity. Bioprinting technology employs precise layer-by-layer deposition to print bio-inks containing endothelial cells, cardiomyocytes, and supportive biomaterials according to predefined structures, enabling directed construction of vascular networks. Research utilizing a 6-axis robotic bioprinter integrated with an oil bath printing system has successfully engineered long-term viable myocardial tissue with rhythmic contractions and capillary networks on vascular scaffolds [80]. However, this technology commonly faces the challenge of balancing the printing performance of bio-inks with cell viability.

It is noteworthy that vascularization construction effectively overcomes the limitations in organoid size and maturity caused by the lack of a vascular system. Specifically, the formation of functional vascular networks not only enlarges the maximum diameter of organoids to hundreds of micrometers [29] but also markedly enhances the survival rate of cells within the core region of organoids [80]. Finally, vascularization also promotes the maturation and differentiation of cardiomyocytes while enhancing the expression levels of relevant maturation markers [81].

Although vascularized organoid technology has developed rapidly, it should be noted that current vascularized cardiac organoid construction still faces enormous limitations. Specifically, it remains challenging to obtain fully functional vascular systems. Existing vascular networks often lack the hierarchical structure comprising small arteries, capillaries, and small veins. The vascular endothelial barrier function is incomplete, and post-transplantation integration with the host vascular system is weak.

### Composite materials integrating with immune components

A recent study demonstrates the potential of novel materials with simulated biological properties to optimize cell seeding techniques and microenvironment reconstruction in cardiac 3D constructs engineering, based on cellular spatial distribution patterns and their interaction dynamics [55]. Previous studies confirm a critical cardiomyocyte–macrophage interaction network in cardiac homeostasis [82]. This system regulates heart metabolism and function, providing new insights for stable cardiac organoid development. The core mechanism involves the binding of damage-associated molecular patterns released by cardiomyocytes to pattern recognition receptors on macrophage surfaces, and the intercellular transmission of mitochondrial quality control signals [82]. Therefore, novel materials could be designed to efficiently promote macrophage mitochondrial phagocytosis. One approach involves modifying the surface of nanomaterials with integrin β1 activation fragments to enhance intercellular signaling [83]. Secondly, by simulating the physical properties of the extracellular matrix, piezoelectric material regulates macrophage polarization and enhances phagocytic activity [84]. In cardiac organoids, these materials may be used to guide macrophages to phagocytose cardiomyocyte-derived exosomes containing defective mitochondria, thereby maintaining mitochondrial and metabolic equilibrium. This approach holds therapeutic potential for mitochondrial cardiomyopathy and related disorders. In the aspect of tissue regeneration, a recent study showed that paracrine signals secreted by iPSC-derived macrophages promote adult cardiomyocyte proliferation [85]; this finding supports the design of composite biomaterials to promote the repair of injured tissues.

Furthermore, integrating immune components including macrophages, pericytes, and supporting biomaterials into vascularized cardiac organoid construction to simulate the in vivo immune–vascular microenvironment represents a crucial developmental direction for creating more physiologically relevant cardiac organoid models. Research has also demonstrated that a 3D extracellular matrix-rich microenvironment promotes cardiomyocyte maturation and enriches cardiac organoids with immunoregulatory signals, thereby enhancing myocardial repair in infarct hearts [29].

In summary, the integration of these advanced biomaterials not only elevates the architectural sophistication of cardiac organoids but also augments their key physiological functionalities, including contractile dynamics, electrophysiological signaling, and pharmacological responsiveness [86,87]. This technological breakthrough establishes a robust, physiologically relevant platform with transformative potential for precision disease modeling, high-throughput drug discovery, diagnostic applications, and regenerative therapies.

## The Application of 3D Cardiac Constructs in Drug Screening

**Fig. 4.**
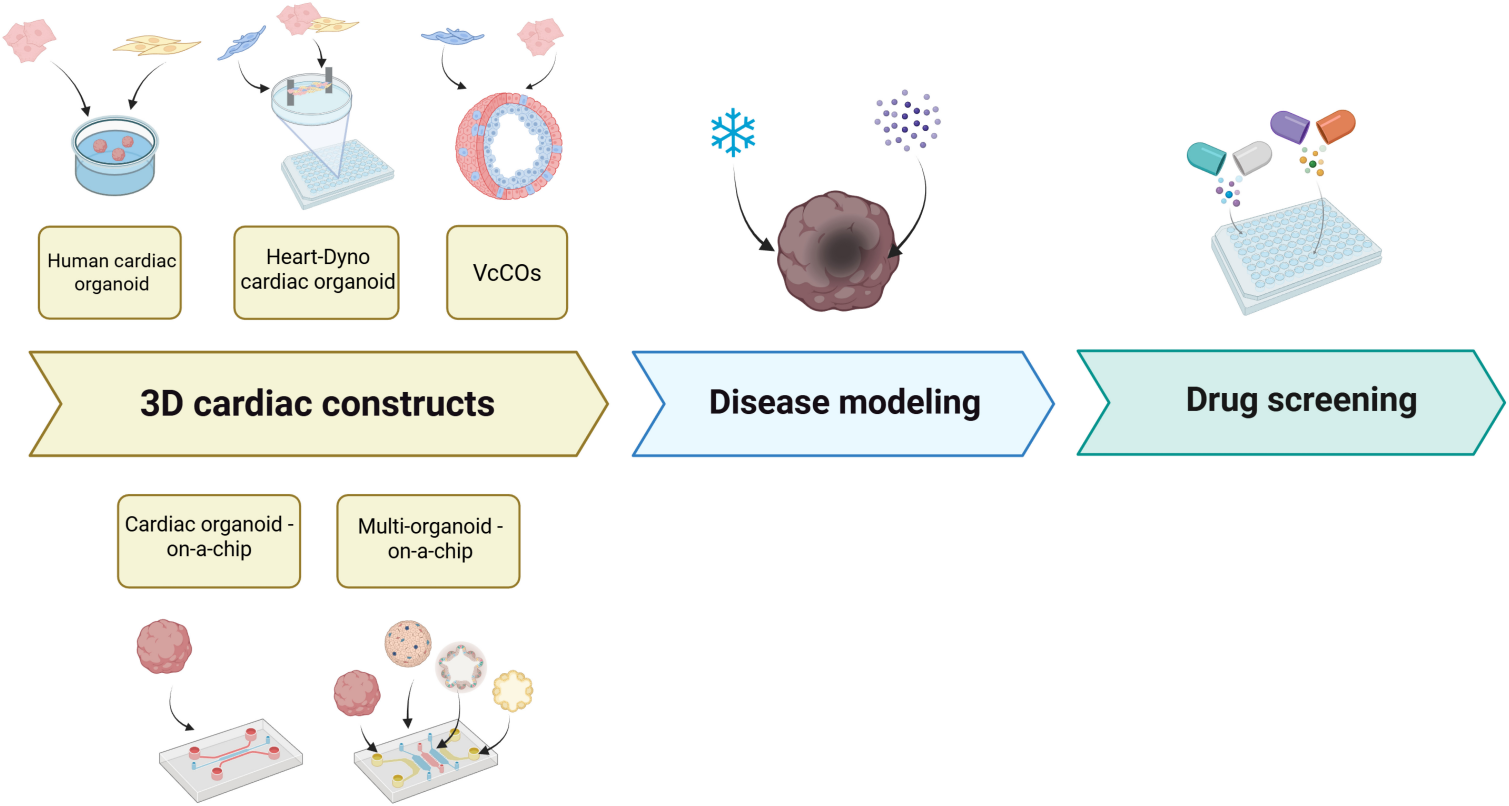
Drug screening in 3D cardiac constructs. This figure illustrates various 3D cardiac constructs utilized in drug screening, highlighting their applications in cardiovascular research and pharmacological testing. VcCOs, vascularized chambered cardiac organoids.

Pharmacological intervention remains one of the most critical approaches for CVD management [88]. Cardiovascular drug screening typically involves testing a library of Food and Drug Administration (FDA)-approved compounds on cardiac cells, such as CFs [89], cardiomyocytes [90], or endothelial cells [91] (Table 4). However, these simplified 2D systems lack the intricate 3D architecture and dynamic cellular cross-talk found in native cardiac tissue [92], thereby reducing the predictive validity of pharmacological outcomes and toxicity assessments. Recent breakthroughs in human 3D cardiac models have facilitated scalable drug screening platforms [27,93–95] (Fig. 4). A study screening 105 potential regenerative compounds (screened out from 5,000 chemical compounds via 2D high-content primary screening experiment) identified 2 that enhanced cardiomyocyte proliferation without impairing rhythm or contractility [50]. Notably, some compounds effective in 2D cultures proved ineffective or detrimental in 3D organoids, demonstrating traditional models’ limitations and organoids’ superior predictive power.

**Table 4.** Drug screening strategies

Type of disease	Drug	Pathological phenotype	Methods	Reference
Venous malformations	Bosutinib	Vascular anomalies	DLEPS (ML)	[91]
DCM	Gö6976 and SB203580	Contractile and metabolic dysfunction	High-throughput screening on 160 well-characterized SMKIs	[90]
Cardiac fibrosis	Artesunate	Collagen deposition	Multiscale drug screening of 5,000 preclinical and clinical compounds (including ML)	[89]
Myocardial infarction	Phased combination of SB431542, Y27632, and CHIR99021	Hypoxic stress, fibrosis, and electrophysiological dysfunction	Seventeen modulators from 9 signaling pathways identified from the RNA-seq analyses	[139]
Brugada syndrome	Quinidine and sotalol	Arrhythmogenesis	Previously reported 4 drugs	[140]
Cardiac arrhythmias	Ruxolitinib	Arrhythmogenesis	4,475 compounds in clinical use	[141]
Pathological cardiac hypertrophy	Luteolin	Cardiac hypertrophy, fibrosis, metabolic disorder, and heart failure	2,570 compounds	[142]
Cardiac fibrosis	Bufalin and lycorine	Fibrosis, decline in diastolic function	480 natural compounds, multiple in vitro fibrosis assays and stringent selection algorithms	[143]

DCM, dilated cardiomyopathy; DLEPS, deep learning-based efficacy prediction system; ML, machine learning; SMKIs, small molecule kinase inhibitors

Recent studies using human cardiac organoids (hCOs) combined with phosphoproteomic and single-nucleus RNA sequencing have identified inflammatory cytokine storms as key contributors to diastolic dysfunction, with interferon-γ, interleukin-1β, and poly(I) as primary mediators [96]. Notably, BET inhibitors like Apabetalone effectively counter SARS-CoV-2-induced cardiac infection and prevent cardiac insufficiency, highlighting their therapeutic potential for COVID-19-related cardiac injury [96]. In a separate model, MI organoids were created using an oxygen diffusion gradient and norepinephrine stimulation [97]. Treatment with JQ1, a BET inhibitor, reduced asynchronous beating and fibrosis, demonstrating MI organoids’ utility as a platform for heart failure drug screening [97]. A recent study investigating myocardial regeneration employed hCOs to screen small molecules targeting calcium cycling proteins in cardiomyocytes. The results revealed that nifedipine, an L-type calcium channel (LTCC) inhibitor, successfully re-induced proliferation in mature cardiomyocytes [98]. Vascularized chambered cardiac organoids (vcCOs) overcome key limitations of traditional organoids by integrating cardiomyocytes, vascular progenitors, endothelial cells, and fibroblasts, enabling functional vascular networks and native-like tissue complexity [99]. Created via co-encapsulation of hiPSC-derived vascular spheres with cardiomyocytes, these 3D constructs exhibit spontaneous contractions and cellular cross-talk. When modeled with cryoinjury, vcCOs recapitulate MI features, while therapeutics like captopril mitigate damage and doxorubicin induces dose-dependent cardiotoxicity (arrhythmias, ATP depletion, and apoptosis). Collectively, these findings demonstrate that hCOs serve as versatile platforms for therapeutic development for CVDs.

Researchers engineered an innovative 3D cardiac model incorporating iPSC-derived cardiomyocytes and CFs to address unpredictable drug toxicity [12]. The system incorporated multiple functional assays to evaluate both FDA-withdrawn (hepatotoxic/cardiotoxic) drugs and safe controls. Compared to 2D cultures, the 3D organoids showed higher sensitivity to cardiotoxins like astemizole, cisapride, mibefradil, and trodifacoum, detecting toxicity at lower doses and earlier time points. Strikingly, observed toxic concentrations matched clinically reported human plasma levels. Importantly, non-toxic drugs at therapeutic doses did not impair organoid contractility or viability, validating their physiological relevance for cardiotoxicity prediction. To enhance its utility, the cardiac organoids were integrated into a multi-organ microplatform (liver, lung, vascular, brain, testis, and colon) [12]. This system maintained long-term tissue viability and biomarker expression, serving as a robust tool for systemic toxicity screening across organ interactions.

3D cardiac models offer great potential for drug screening but face limitations in replicating dynamic physiological conditions. Static 3D cultures lack fluidic interactions, which are crucial for modeling micro-physiological environments [100]. Organ-on-a-chip platforms address this by integrating organoid technology with microfluidics, creating perfusable chambers that replicate the 3D architecture and physiological functions of human organs. These systems enhance drug development and toxicity studies by simulating in vivo conditions through controlled flow and nutrient exchange [101]. By developing microfluidic multi-cellular heart-on-chip models, drug permeability has been improved, and the pathological phenotypes observed are more consistent with in vivo conditions [10,11]. Using organ-on-chip technology, it is possible to simultaneously assess the hepatic metabolism of cardiac drugs and evaluate the cardiotoxicity of antineoplastic drugs [102], as well as the impact of environmental factors on multiple organs, including the heart [103].

Using stem or patient-derived cells, they self-assemble into functional organ-like units while microfluidics enables precise regulation of nutrient delivery, mechanical forces, and biochemical gradients, essential for studying dynamic pathologies and drug responses. Organoids-on-a-chip exhibit 3 key advancements: (a) enhanced physiological fidelity, accurately replicating tissue interfaces and cell–cell interactions; (b) versatile construct design, allowing assembly from either stem/progenitor cell differentiation or patient biopsies for personalized drug testing; and (c) scalability, supporting high-throughput integration for efficient screening. Moreover, multi-organoid integrated systems demonstrate superior capability in detecting drug toxicity post-hepatic metabolism, owing to the incorporation of functional liver organoids [12]. This is particularly critical for prodrugs like cyclophosphamide, which are inherently non-toxic but metabolize into harmful compounds, a process the platform reliably replicates to reflect clinical outcomes [12]. The micro-physiological system-on-a-chip platform (MSCP) addresses limitations of conventional preclinical models by integrating pneumatic microvalves and a 304-well array, enabling dynamic switching between “organ formation” and “blood flow” modes [102]. This system supported a functional 4-organ model (heart, liver, intestines, and lung cancer spheroids). Evaluation of 4 anti-lung cancer drugs (doxorubicin, cisplatin, docetaxel, and pemetrexed) revealed the following: (a) universal normal organ toxicity; (b) severe cardiotoxicity from docetaxel/doxorubicin (reduced cardiac spheroid viability); and (c) attenuated antitumor efficacy due to intestinal/hepatic drug metabolism. The MSCP uniquely combines high-throughput multi-organ modeling with physiological inter-organ communication simulation and concurrent efficacy/toxicity assessment (particularly cardiotoxicity) [102]. Collectively, this platform demonstrates transformative potential for improving drug safety prediction and personalized medicine approaches.

Traditional static cell cultures and electrophysiological methods (e.g., microelectrode arrays and patch-clamp) struggle to replicate the heart’s 3D microenvironment, limiting their predictive accuracy for drug screening. To address this, researchers have introduced the Heart-on-a-Miniscope, an organoid-chip system combining microfluidics with a miniaturized fluorescence microscope [104]. The key innovations include the following: (a) Optical redesign: The open-source miniscope was modified to achieve long working distance (LWD-Miniscope), enabling high-resolution calcium imaging through ~100-μm glass chips. (b) Integrated stimulation: A 7-microwell chip with carbon rod electrodes delivers programmable electrical pacing for precise beat frequency control. (c) Pathological modeling: Successful recapitulation of Ivabradine-induced beating suppression demonstrated frequency-dependent drug evaluation. The platform detected the cardiotoxic effects of compounds (e.g., dofetilide and quinidine) by analyzing calcium transient kinetics (TTP, CD90, and Decay50/30), showcasing robust toxicity assessment capabilities. This cost-effective system integrates real-time 3D optical imaging, electrophysiological modulation, and organoid-based physiological modeling, thereby enhancing preclinical cardiotoxicity screening and enabling early-stage compound risk assessment.

Regarding practical implementation of cardiac 3D constructs, there are still several challenges that remain. In terms of cost, 3D models have higher initial construction and culture expenses than the conventional 2D culture system but remain relatively more economical than animal models [105]. Its upfront costs are markedly higher than simple 2D culture, involving stem cell maintenance, 3D matrices, growth factors, and potential functional readout equipment [106]. However, cardiac 3D models enable earlier and more accurate detection of cardiotoxicity or lack of efficacy, potentially reducing downstream costs associated with late-stage clinical failures [12,50].

Second, throughput and scalability are core bottlenecks for drug screening in current technological development. Traditionally manual cardiac organoid culture suffers from low throughput and high variability, making it unsuitable for large-scale compound library screening. Scalability has been improved through platforms such as 96-well plates for medium-throughput screening, and automation coupled with microfluidic organ-on-chip systems is further enhancing throughput and reproducibility [101,107]. However, achieving industrial-scale ultra-high-throughput screening requires further breakthroughs in organoid production standardization and data analysis pipeline.

The experimental period is also an important influencing factor. A typical experiment cycle includes several weeks to a month for hiPSC differentiation into cardiomyocytes and organoid maturation, followed by several days to a week of drug treatment and phenotypic analysis. The entire process usually takes 1 to 2 months, which is shorter than the multi-month cycle required to establish animal disease models. While longer than simple 2D cell viability assays that can be completed in days, organoids provide multi-dimensional data integrating structure and cellular heterogeneity, offering far more information than 2D endpoint assays [108].

## Digitization of 3D Models and the Roles of Artificial Intelligence

While cardiac 3D models have proven invaluable for drug screening, their scalability and data-driven optimization demand integration with artificial intelligence (AI) to facilitate next-generation precision medicine. Current cardiac 3D model research faces key limitations: (a) Matrix-dependent culture systems with variable growth factor formulations introduce batch inconsistencies, potentially compromising reproducibility. (b) Static imaging paradigms lack temporal resolution to capture dynamic processes like cell migration/differentiation. (c) Light scattering obscures 3D architecture while manual microscope adjustments prevent volumetric high-resolution reconstruction [109]. To address these issues, AI technology is deeply integrated into cardiac organoid research, providing powerful innovative tools for high-throughput phenotypic assessment, deep mechanism analysis, model parameter optimization, and intelligent imaging analysis.

AI technology demonstrates exceptional efficiency in rapidly quantifying complex cardiac 3D pathological models through computer vision and machine learning. One study systematically extracted over 110 high-dimensional features from numerous control and inflammatory cardiac organoids [110]. This research combined automated image segmentation and feature extraction with brightfield microscopy videos. The resulting inflammation scoring model completes the entire workflow from video acquisition to quantitative assessment within 30 min, demonstrating close correlation with molecular biomarker levels.

Beyond rapid evaluation of complex pathological phenotypes, AI plays a pivotal role in deciphering the underlying molecular mechanisms of cardiac development and disease. For instance, in a study investigating NKX2-5 deficiency [111], researchers established atrioventricular-specific cardiac organoid models and obtained high-resolution single-cell RNA sequencing data. To address the subjectivity and low throughput of manual annotation, the team developed a machine learning label transfer method based on random forests. Using annotated human fetal heart single-cell data as the training set, this method successfully achieved automated and precise annotation of organoid cell types, chamber identity, and left–right lateralization. Applying this model to analyze organoids with the NKX2-5 c.673C>A mutation revealed a marked presence of cells predicted as atrial identity within the ventricular lineage, precisely quantifying the pathological defect of “atrialization of ventricles”. This study demonstrates AI’s powerful capabilities in uncovering cell lineage defects in cardiac organoid disease models.

Moreover, AI technology has been applied to guide the rational design and functional optimization of cardiac organoid construction. One study integrated micro-patterning technology with machine learning to construct a library of 230 cardiac organoids featuring 7 geometric shapes, extracting 10 key physiological parameters [112]. Subsequently, supervised learning models identified calcium transient rise time as the primary feature distinguishing functional impacts of different geometric designs, suggesting that geometric constraints primarily act by modulating calcium handling capacity. Direct experimental validation confirmed that the AI-predicted superior rectangular 1:4 organoids exhibited enhanced sarcomere alignment, calcium handling gene expression, and contraction coordination. This study demonstrates AI’s value in deciphering structure–function relationships and guiding rational organoid design. Another integrated approach is demonstrated by the AI analysis software Organalysis [113]. This model incorporates machine learning modules to automatically analyze high-throughput images and quantify the relative importance of culture conditions on cardiac organoid formation. Therefore, this AI-driven model can also be used to optimize cardiac organoid construction protocols.

Traditional imaging and quantification methods for cardiac organoids heavily rely on physical fluorescent labeling and manual analysis, suffering from limitations such as invasiveness and low throughput. Generative AI offers novel solutions for label-free cell identification. For instance, researchers successfully trained a conditional GAN model capable of directly converting brightfield images of cardiac organoids into virtual fluorescent images specific to cardiovascular cell types, enabling label-free cell identification and quantification [114]. This application demonstrates that AI is expanding from backend data analysis into frontend experimental image generation, comprehensively enhancing organoid research.

Since high-throughput screening requires large quantities of cells, an increase in throughput also leads to higher screening costs. Because of the high production cost of cardiac organoids, drug screening is still primarily conducted at the cellular level in many cases. Thus, reducing the cost of drug screening has become a major challenge. AI-assisted drug screening can improve screening accuracy by predicting drug efficacy based on ADMET (absorption, distribution, metabolism, excretion, and toxicity) [89,115]. Additionally, AI-assisted digital screening can be performed using RNA sequencing data to analyze pathway alterations, thereby narrowing down compound candidates for functional screening and reducing overall screening costs [91,116]. Furthermore, imaging-based AI can be applied in digital drug screening for cardiac fibrosis [117]. In this approach, fibrosis image features are extracted for machine learning-based classification to determine the presence of fibrosis. Specifically, a linear classifier can be trained to identify the fibrogenic phenotype from microscope images. This machine learning algorithm processes over 100 extracted features from each analyzed cell, resulting in an optimized linear separation between phenotypic groups [118]. Currently, the primary application of organoids in drug discovery is to evaluate the efficacy of a limited number of drugs. However, by integrating AI-assisted drug screening, researchers can narrow the scope of drug efficacy testing and greatly enhance drug development efficiency.

AI-assisted research on non-cardiac organoids also provides valuable insights for technology development. For instance, in drug-induced liver injury (DILI) level prediction, the DILITracer model employs the BEiT-V2 pre-trained network to analyze spatiotemporal features in brightfield liver organoid images [119]. Integrating an image–space–time encoding layer enhances multidimensional data fusion, achieving 82.34% accuracy in ternary DILI classification (no, low, or high risk). Notably, it identifies non-DILI compounds with 90.16% accuracy. The reported overall accuracy was derived from the model’s performance on the test set split from its primary dataset, evaluated using 5-fold cross-validation with an 80:20 train–test split applied within each fold, ensuring a robust internal evaluation.

Accurate cell segmentation in organoid imaging is crucial for long-term in vivo studies and high-throughput screening. AI-driven tools, such as the Cellos platform, combine classical image processing with the StarDist-3D Convolutional Neural Networks to segment organoid cells/nuclei efficiently [120]. In triple-negative breast cancer organoids, it achieved 96.07% accuracy, enabling quantification of cisplatin-induced dynamic changes in volume, nuclear morphology, and spatial cell interactions [120]. The reported accuracy was validated by manually evaluating 321 segmented organoids from cisplatin-treated wells, and the model’s generalizability was further confirmed on 3 external datasets: published breast cancer spheroids, HL60 leukemia synthetic images, and a mouse embryo image, demonstrating cross-sample applicability. In summary, these AI-driven studies offer valuable insights for organoid technical improvement through digital transformation and AI integration.

Although AI-assisted cardiac organoid research is booming, there are still barriers restricting the AI-based analysis across different laboratories. First, variations in organoid culture protocols, imaging systems, and acquisition parameters lead to notable data distribution shifts. Furthermore, inconsistent fluorescent labeling strategies and cell-type annotation criteria hinder the creation of harmonized datasets necessary for robust multi-center validation. Finally, there are no universally accepted benchmark datasets or validation protocols for comparing AI performance in organoid analysis, making cross-study comparisons and clinical validation difficult.

In summary, cardiac organoids combined with AI and nanofabrication technologies are exerting growing influence on drug development strategies, laying the foundation for next-generation screening approaches that are more economical and physiologically accurate.

## Limitations and Potential of 3D Cardiac Constructs Technology

Despite considerable advancements in 3D cardiac constructs development, their application and reliability remain limited due to technical barriers. These limitations include variability in iPSC induction efficiency and differentiation [5], heterogeneity in cardiomyocyte and non-cardiomyocyte ratios affecting functional readouts [48], the need for Good Manufacturing Practices (GMP)-compliant scalable production [121], and the current resource-intensive nature of organoid generation. Addressing these aspects is critical for advancing organoids toward drug discovery and clinical applications.

One big challenge involves the formation of cardiac organoids using iPSC-EC, iPSC-CM, and iPSC-CF within a shared culture medium. However, such universal culture media often fail to replicate the special microenvironment required by each cell type. This results in a loss of cell-type specificity, as growth factors, metabolites, and small molecules cannot be tailored to the needs of the individual cells. Another major challenge is the integration of iPSC-derived cardiac tissues into host hearts. Poor electromechanical integration often leads to incomplete synchronization between the graft and the host, which may result in arrhythmias and conduction blocks. This limitation is likely due to the immaturity of organoids, which currently exhibit simplified tissue organization and electrical functionality of the adult heart. Advanced tissue engineering techniques, such as scaffolding, electrical pacing, and vascularization strategies, may enhance tissue maturation and improve compatibility with host organs. Additionally, personalized transplantation strategies using patient-specific iPSCs could enhance transplantation success rates and reduce the risks of immune rejection.

Cardiomyocyte immaturity represents one of the most critical limitations restricting the translational relevance of cardiac organoids. hPSC-CMs generally resemble a fetal or neonatal state rather than adult myocardium. Immature cardiomyocytes are characterized by disorganized sarcomeres with incomplete α-actinin alignment, reduced sarcomere length, absence or poor development of T-tubules, smaller cell size, and a predominantly mononucleated phenotype [122]. At the metabolic level, immature cardiomyocytes rely mainly on glycolysis, whereas adult cardiomyocytes undergo a postnatal metabolic switch toward fatty acid oxidation accompanied by mitochondrial biogenesis and transcriptional remodeling [123]. These structural, electrophysiological, and metabolic features collectively distinguish immature organoid cardiomyocytes from adult human heart tissue.

To address this limitation, multiple maturation strategies have been developed. Electrical pacing enhances action potential propagation, calcium handling, and sarcomere organization [124], while mechanical loading or cyclic stretch promotes structural alignment, increased cell size, and improved contractile performance [125]. Prolonged culture duration allows gradual maturation [126] but is often insufficient on its own. Hormonal stimulation, particularly with triiodothyronine (T3) and glucocorticoids such as dexamethasone, accelerates structural and electrophysiological maturation [127]. Importantly, metabolic maturation media that reduce glucose and insulin while promoting fatty acid availability have been shown to drive the metabolic switch to fatty acid oxidation, induce mitochondrial maturation, and promote cardiomyocyte cell cycle exit [128]. These findings highlight that distinct aspects of cardiomyocyte maturation, including structural, electrophysiological, and metabolic properties, are regulated by partially independent cues and require combinatorial optimization.

Cardiomyocyte maturation status has direct consequences for drug response prediction and cardiotoxicity assessment. Immature cardiomyocytes exhibit altered ion channel expression, calcium handling, β-adrenergic signaling, and metabolic enzyme profiles, which can lead to inaccurate prediction of drug efficacy and arrhythmogenic or cardiotoxic effects [129]. For example, immature models may underestimate pro-arrhythmic risk due to incomplete repolarization reserve or misrepresent metabolic drug toxicity, because of their reliance on glycolysis rather than oxidative metabolism [130]. Enhanced maturation improves concordance with adult human cardiac responses, thereby increasing the reliability of pharmacological screening, safety pharmacology, and disease modeling. Consequently, improving cardiomyocyte maturation is essential for advancing cardiac organoids from developmental models toward clinically predictive platforms.

Several key areas for improvement and future opportunities include the following: (a) Enhancing imaging depth and resolution; currently, whole-mount imaging of organoids is limited to the outer layers, while imaging of the organoid center often requires frozen sectioning, increasing experimental time and complexity. The development of confocal microscopy with higher resolution and imaging depth could substantially advance organoid research. Moreover, real-time imaging in fluorescent reporter organoids will require next-generation imaging systems with higher resolution and sensitivity. (b) Another area of improvement is establishing functional vascular networks, as most organoids lack self-organized vascular networks, leading to inadequate nutrient supply in deeper layers, difficulty in long-term culture, and poor permeability of viral vector-based drugs. Developing efficient vascularization strategies would greatly enhance organoid viability and enable more physiologically relevant models. (c) Furthermore, developing functional measurement platforms and standardized assays is critical, as most commonly used experimental platforms are designed for 2D culture systems, which do not fully support functional assessments of 3D cardiac organoids. There is also a lack of standardized functional parameter sets for measuring electrophysiology, calcium signaling, and contractility in organoids. The development of 3D-compatible functional measurement platforms and standardized benchmarking assays is urgently needed. (d) Additionally, large batch-to-batch and donor-to-donor variability is frequently observed in organoids derived from different cell sources, requiring more robust and standardized 3D culture strategies to improve reproducibility and reliability in organoid-based research.

## Conclusion

With continuous advancements in technology, cardiovascular organoids are becoming increasingly complex and powerful. Currently, cardiac organoids have demonstrated the ability to produce blood [131], and it is foreseeable that in the future, autonomous blood circulation between multiple organoids will be achievable. Multichambered cardiac organoids can model distinct chamber-specific responses to drugs [18,93]. Additionally, constructing microfluidic 3D multicellular heart-on-chip models can enhance nutrient supply, physiological complexity, and drug penetration [11]. Recent studies on heart-on-chip technology have highlighted the contribution of primitive macrophages to sustained vascular network formation in myocardial tissue [132]. Moreover, co-culturing multiple cell types has been shown to increase tissue complexity, allowing for a more realistic simulation of pathological phenotypes in heart-on-chip models [10,11]. In 3D cardiac microtissues, stromal cells derived from human iPSC have demonstrated the critical roles of non-cardiomyocytes in heart disorders such as fibrosis and dysfunction [24]. Therefore, the development of multicellular organoids is essential for recapitulating in vivo organ functions.

Despite the tremendous progress in cardiovascular organoid technology, further refinements are needed. In diseases such as COVID-19, inflammatory responses are key contributors to cardiac pathogenesis. To replicate the inflammatory storm caused by COVID-19 infection, researchers have used inflammatory factors to induce pathological responses in cardiac organoids, leading to diastolic dysfunction [96]. Studies on heart-on-chip models containing primitive macrophages have also revealed the indispensable contribution of immune cells in the development and maturation of cardiac tissue [132]. However, the immunoregulatory function of tissue-resident mature macrophages in organoids remains largely unknown. There is no doubt that cardiac organoids incorporating mature cardiac-resident macrophages will become an essential tool for studying cardiovascular homeostasis.

While 3D constructs offer great potential for modeling human cardiac diseases and testing therapies, their path to regulatory acceptance by agencies like the FDA (USA) or European Medicines Agency (Europe) remains unclear. Key challenges include establishing reproducibility standards across labs, ensuring scalability under GMP for clinical-grade production, and addressing cost barriers to make 3D constructs viable for large-scale drug screening or personalized medicine. Improvements on these aspects, such as regulatory alignment, cost-effective protocols, and scalable manufacturing, would strengthen this technology for applications in drug development and patient-specific therapy.

To advance the field of cardiac 3D models, a structured roadmap with clear milestones is essential. In the near term, the focus should be on refining protocols for generating standardized, scalable cardiac 3D constructs with enhanced vascularization and immune cell integration. These advancements will enable high-fidelity modeling of complex cardiac pathologies, such as arrhythmias and environmental-induced cardiomyopathies, positioning organoids as powerful tools for drug toxicity screening and mechanistic studies. Emerging technologies like spatial omics could revolutionize this landscape by enabling single-cell resolution mapping of cardiac organoid microenvironments, revealing how cell–cell interactions drive disease phenotypes. Similarly, comprehensive databases linking organoid models to patient-specific genomic and clinical data may promote predictive modeling of individual responses to therapies. Clinical translation is expected within 5 to 10 years, with initial applications in personalized medicine. Importantly, 3D cardiac constructs should not replace but complement existing preclinical animal studies, offering unique advantages in human relevance. By integrating 3D cardiac constructs into the broader preclinical applications, researchers can accelerate discoveries while maintaining ethical and practical feasibility.

In summary, the future development of 3D cardiac constructs technology is expected to focus on (a) achieving functional interactions between multiple organ systems; (b) constructing organoids with enhanced vascularization and immune cell integration; (c) generating 3D models with increased maturity and structural diversity; and (d) establishing standardized detection parameter sets and consensus protocols for scalable manufacturing. As research in this field continues to advance, cardiac 3D constructs with diverse functions and capabilities will continue to emerge, engineering physiologically relevant in vitro platforms for preclinical drug testing and drug discovery.

### Search strategies

A systematic literature search was conducted in PubMed and the Web of Science Core Collection database. Keywords include cardiac organoid, cardiac microtissues, EHT, heart on chip, vascularized cardiac organoids, biomaterials, artificial intelligence, and drug screening. Inclusion criteria focused on studies addressing construction methods, functional evaluation systems, material innovations, or clinical translation potential for 3D cardiac constructs. Document types were restricted to original research papers and systematic reviews.
